# Supplementary material for: Dextran-based T-cell expansion nanoparticles for manufacturing CAR T cells with augmented efficacy
Source: Nat Commun. 2026 Jan 20;17:1103. doi: 10.1038/s41467-025-67868-1 (PMC12852890; doi:10.1038/s41467-025-67868-1)
Supplement: Supplementary file 2 — Description of Additional Supplementary File [file 41467_2025_67868_MOESM2_ESM.pdf]

Description of Additional Supplementary Files

Supplementary Movie 1: Airyscan imaging (3D view) on how T-Expand specifically interacting in engineered Jurkats

Supplementary Movie 2: Airyscan imaging (3D view) on how DynabeadsTM specifically interacting in engineered Jurkats
