# Supplementary material for: Dextran-based T-cell expansion nanoparticles for manufacturing CAR T cells with augmented efficacy
Source: Nat Commun. 2026 Jan 20;17:1103. doi: 10.1038/s41467-025-67868-1 (PMC12852890; doi:10.1038/s41467-025-67868-1)
Supplement: Supplementary file 5 — Reporting Summary [file 41467_2025_67868_MOESM5_ESM.pdf]

nature portfolio

Corresponding author(s): Yi Sun, Sine Reker Hardup

Last updated by author(s): Dec 3, 2025

Reporting Summary

Nature Portfolio wishes to improve the reproducibility of the work that we publish. This form provides structure for consistency and transparency in reporting. For further information on Nature Portfolio policies, see our Editorial Policies and the Editorial Policy Checklist.

Statistics

For all statistical analyses, confirm that the following items are present in the figure legend, table legend, main text, or Methods section.

n/a	Confirmed	
☐	☒	The exact sample size (n) for each experimental group/condition, given as a discrete number and unit of measurement
☐	☐	A statement on whether measurements were taken from distinct samples or whether the same sample was measured repeatedly
☐	☒	The statistical test(s) used AND whether they are one- or two-sided<br>*Only common tests should be described solely by name; describe more complex techniques in the Methods section.*
☐	☐	A description of all covariates tested
☐	☐	A description of any assumptions or corrections, such as tests of normality and adjustment for multiple comparisons
☐	☐	A full description of the statistical parameters including central tendency (e.g. means) or other basic estimates (e.g. regression coefficient) AND variation (e.g. standard deviation) or associated estimates of uncertainty (e.g. confidence intervals)
☐	☒	For null hypothesis testing, the test statistic (e.g. F, t, r) with confidence intervals, effect sizes, degrees of freedom and P value noted<br>*Give P values as exact values whenever suitable.*
☒	☐	For Bayesian analysis, information on the choice of priors and Markov chain Monte Carlo settings
☒	☐	For hierarchical and complex designs, identification of the appropriate level for tests and full reporting of outcomes
☒	☐	Estimates of effect sizes (e.g. Cohen's d, Pearson's r), indicating how they were calculated

Our web collection on statistics for biologists contains articles on many of the points above.

Software and code

Policy information about availability of computer code

Data collection	BD LSRFortessa™ Cell Analyzer, Incucyte® Sartorius, Optical Imaging unit- MILabs
Data analysis	Statistical analysis was performed in GraphPad version-10,<br>CAR T killing assay and re-challenge assay was performed in Incucyte® Sartorius live cell image analysis software,<br>Flow cytometry analysis was performed using FolwJo v10<br>Cell imaging figure was processed by Imaris software (v.10.1.0).

For manuscripts utilizing custom algorithms or software that are central to the research but not yet described in published literature, software must be made available to editors and reviewers. We strongly encourage code deposition in a community repository (e.g. GitHub). See the Nature Portfolio guidelines for submitting code & software for further information.

Data

Policy information about availability of data

All manuscripts must include a data availability statement. This statement should provide the following information, where applicable:

- Accession codes, unique identifiers, or web links for publicly available datasets
- A description of any restrictions on data availability
- For clinical datasets or third party data, please ensure that the statement adheres to our policy

The data supporting this study are provided in the main manuscript and the Supplementary Information. Additional data may be obtained from the corresponding author upon reasonable request. Source data are provided with this paper.

Research involving human participants, their data, or biological material

Policy information about studies with human participants or human data. See also policy information about sex, gender (identity/presentation), and sexual orientation and race, ethnicity and racism.

Reporting on sex and gender	No such information has been gathered.
Reporting on race, ethnicity, or other socially relevant groupings	No such information has been gathered.
Population characteristics	No such information has been gathered.
Recruitment	Human peripheral blood was obtained from healthy adults after obtaining informed consent (Technical University of Denmark - Rigshospitalet National Hospital approval BC-40). No personal information was gathered, and donors were anonymized for this study. All T cells were derived from the blood of healthy donors collected at the central blood bank at Rigshospitalet (Copenhagen, Denmark).
Ethics oversight	This study was carried out in accordance with the Declaration of Helsinki, and under the agreement: Technical University of Denmark - Rigshospitalet National Hospital approval BC-40.

Note that full information on the approval of the study protocol must also be provided in the manuscript.

Field-specific reporting

Please select the one below that is the best fit for your research. If you are not sure, read the appropriate sections before making your selection.

☒ Life sciences ☐ Behavioural & social sciences ☐ Ecological, evolutionary & environmental sciences

For a reference copy of the document with all sections, see nature.com/documents/nr-reporting-summary-flat.pdf

Life sciences study design

All studies must disclose on these points even when the disclosure is negative.

Sample size	Sample sizes for animal studies were determined based on currently available literature and experience with xenograft models. Four mice were included per group. For experiments not involving animals, sample size calculations were not performed. Based on our past experience with inter-donor variability, we selected samples from approximately 3 to 5 healthy donors.
Data exclusions	All collected data were included in the analysis except for one healthy donor, whose T cell expansion failed for reasons unrelated to our nanoparticle platform. This donor was therefore excluded from downstream analyses. No other data exclusions were performed, and this exclusion did not affect the overall conclusions
Replication	The experiments were repeated as described in the figure captions.
Randomization	Mice were randomly assigned to experimental groups after tumor inoculation and before CAR-T cell administration, and were subsequently treated with CAR-T cells expanded using either T-Expand or Dynabeads. For studies not involving animals, randomization was not required, as each sample corresponded to a uniquely identified healthy donor and was processed with all applicable conditions (e.g., T-Expand, TransAct, or Dynabeads).
Blinding	Blinding was not applied during the animal studies, as investigators administered CAR-T cell injections with inherent variability between injections. For studies not involving animals, blinding was not applicable.

Reporting for specific materials, systems and methods

We require information from authors about some types of materials, experimental systems and methods used in many studies. Here, indicate whether each material, system or method listed is relevant to your study. If you are not sure if a list item applies to your research, read the appropriate section before selecting a response.

Materials & experimental systems

n/a	Involved in the study
☐	☒ Antibodies
☐	☒ Eukaryotic cell lines
☒	☐ Palaeontology and archaeology
☐	☒ Animals and other organisms
☒	☐ Clinical data
☒	☐ Dual use research of concern
☒	☐ Plants

Methods

n/a	Involved in the study
☒	☐ ChIP-seq
☐	☒ Flow cytometry
☒	☐ MRI-based neuroimaging

Antibodies

Antibodies used

anti-human CD3 BioXcell OKT-3 (BE0001-2)
anti-human CD28 BioXcell 9.3 (BE0248)
BB700-anti-human CD25 (IL-2R) BD Biosciences M-A251 (566447)
BV786-anti-human CD69 BD Biosciences FN50 (563834)
PE-anti-human CD69 Biolegend FN50 (310906)
BV421-anti-human CD3 BD Biosciences SP34-1 (562877)
BV711-anti-human CD45RA BD Biosciences HI100 (563733)
APC-anti-human CD197 (CCR7) BD Biosciences 2-L1-A (566762)
PE/Cy7-anti-human TIM-3 Biolegend F38-2E2 (345014)
BUV737-anti-human CD279 (PD-1) BD Biosciences EH12.1 (612791, 565299)
Validation
BV650-anti-human CD223 (LAG-3) BD Biosciences 11C3C65
(369316)
PE-Cy7-anti-human CD57 Biolegend QA17A04 (393310,
B323195)
BV786-anti-human CD39 BD Biosciences TU66 (742523)
BV605-anti-human TIGHT BD Biosciences 741182 (RUO) (747841)
BV480-anti-human CD4 BD Biosciences L200 (566148)
PerCP-anti-human CD8 BD Biosciences SK1 (347314)
BV480-anti-human CD8 BD Biosciences RPA-T8 (566121)
BUV737-anti-human CD28 BD Biosciences CD28.2 (612815)
BV605-anti-human CD27 Biolegend O323 (302830)
PE-anti-human CD137 BD Biosciences C65-485 (RUO)
BV711-anti-CD19 BD Biosciences SJ25C1 (563036)
PE-Cy7-anti-CD4 BD Biosciences RPA-T4 (560649)
BV421 CD25 IL-2R M-A251 BD (562442)

Validation

Validation and citations for the common usage can be found for the individual antibodies on the vendors website. Antibodies were titrated to find the optimal staining concentration.

Eukaryotic cell lines

Policy information about cell lines and Sex and Gender in Research

Cell line source(s)	ATCC (American type culture collection)
Authentication	Authentication and associated testing was performed by ATCC.
Mycoplasma contamination	The cell lines were checked periodically for mycoplasma and all the cell lines used in this study was mycoplasma negative
Commonly misidentified lines (See ICLAC register)	No commonly misidentified cell lines were used.

Animals and other research organisms

Policy information about studies involving animals; ARRIVE guidelines recommended for reporting animal research, and Sex and Gender in Research

Laboratory animals	For in vivo studies, 6-week-old female NXG Immunodeficient mice (NOD-Prkdcscid-IL2rgTm1/Rj) were acquired from Janvier Labs and housed at the Bio Facility, Department of Health Technology, Technical University of Denmark.
Wild animals	N/A
Reporting on sex	Female NXG mice were used for efficacy studies
Field-collected samples	No field collected samples were used in the study.
Ethics oversight	For in vivo studies, 6-week-old female NXG Immunodeficient mice (NOD-Prkdcscid-IL2rgTm1/Rj) were acquired from Janvier Labs and housed at the Bio Facility, Department of Health Technology, Technical University of Denmark. The mice were housed separately from the immunocompetent mice. The mice were housed in groups of 4-5 mice per cage, and the feeding was replenished every 3 days or as required. All procedures were approved by the Danish National Animal Experiment Inspectorate and the institutional ethical board(Approval no. 2020-15-0201-00748).

Note that full information on the approval of the study protocol must also be provided in the manuscript.

Plants

Seed stocks	*Report on the source of all seed stocks or other plant material used. If applicable, state the seed stock centre and catalogue number. If plant specimens were collected from the field, describe the collection location, date and sampling procedures.*
Novel plant genotypes	*Describe the methods by which all novel plant genotypes were produced. This includes those generated by transgenic approaches, gene editing, chemical/radiation-based mutagenesis and hybridization. For transgenic lines, describe the transformation method, the number of independent lines analyzed and the generation upon which experiments were performed. For gene-edited lines, describe the editor used, the endogenous sequence targeted for editing, the targeting guide RNA sequence (if applicable) and how the editor was applied.*
Authentication	*Describe any authentication procedures for each seed stock used or novel genotype generated. Describe any experiments used to assess the effect of a mutation and, where applicable, how potential secondary effects (e.g. second site T-DNA insertions, mosiacism, off-target gene editing) were examined.*

Flow Cytometry

Plots

Confirm that:

- ☒ The axis labels state the marker and fluorochrome used (e.g. CD4-FITC).
- ☒ The axis scales are clearly visible. Include numbers along axes only for bottom left plot of group (a 'group' is an analysis of identical markers).
- ☒ All plots are contour plots with outliers or pseudocolor plots.
- ☒ A numerical value for number of cells or percentage (with statistics) is provided.

Methodology

Sample preparation	For tetramer and surface marker staining, cells were centrifuged at 500 x g for 5 minutes at 4°C, and the supernatant was discarded. The pelleted cells were then resuspended in 5 µL of 1 µM Dasatinib (LC Laboratories, #D3307), antigen tetramers were added, and the cells were incubated for 15 minutes at 37°C in the dark. The cells were washed once with FACS buffer, followed by staining with Near-IR 28 (NiR) viability dye (Invitrogen, #L34976) and additional antibodies for surface staining for surface markers 30 min at 4°C in the dark. Finally, the cells were washed twice with FACS buffer in 200-300 µL of FACS buffer and immediately analysed on a LSRFortessa flow cytometer (BD, USA). Alternatively, the cells were fixed with 50 µL of 1% paraformaldehyde for 1-2 hrs (if required), washed twice with FACS buffer, and analysed 2-24 hrs later.
Instrument	LSRFortessa flow cytometer (BD, USA)
Software	Gating and compensation: - FlowJo™ v10.8 Software
Cell population abundance	For phenotyping of the expanded T cell cultures:<br>200000 events were collected per replicate and 100000-80000 were live 'lymphocytes' of which 100000-

80000 were CD3+T cells and 60000-20000 events were CD19 CAR T cells if the cultures were transduced.
For proliferation assay of T cells expanded and transduced using T-Expand and DynabeadsTM:
200000 events were collected per replicate of which 100000-50000 were live CD3+ T lymphocytes and 40000-10000 events were CD19 CAR T cells.
For analysis of the cells from spleen and bone marrow of the mice treated with T cells or CAR T cells expanded with T-Expand or Dynabeads
For Spleen 1500000 events were collected per mice and about 800000 -500000 events were lymphocytes of which 2000-3000 events were CD3+ Human T cells in the Dynabeads expanded cohort and 2000-10000 events were CD3+ Human T cells in the T-Expand expanded cohort.
For bone marrow 3000000 events were collected per mice and about 1600000 -1000000 events were lymphocytes of which 100-300 events were CD3+ Human T cells in the Dynabeads expanded cohort and 100-10000 events were CD3+ Human T cells in the T-Expand expanded cohort.
For analysis of T cells after three rounds of target cell rechallenge in cytotoxicity assay.
100000-50000 events were recorded for each replicate of which 80000-30000 events were live 'lymphocytes' and 10000-25000 events were CD3+ T cells for Dynabead expanded cohort and 30000-50000 were CD3+ T cells for T-Expand cohort

Gating strategy

The gating strategy employed in the proliferation experiment as follows: First the 'Lymphocytes' were gated SSC-A:FSC-A to remove cellular debri; further the 'singlets' were gated FCS-A:FSC_H and the live dead were dated in SSC-A: Near-infrared dye. The live cells are then gated for CD3+T cells (SSC-A:CD3) and CD19 CAR (SSC-A:CD19 TETRAMER).Finally the Cell Trace violet expression of the CD19 CAR T+T cells is gated in (SSC-A:BV421). On day 0 the cells were acquired to gate the cell trace violet expression of the un-divided T cells. Proliferation index plug-in of the FlowJo™ v10.8 Software was used to calculate the peaks to predict the number of division using the un-divided T cell gate (Histogram:Cell Trace BV421) as a control.
Gating strategy for T cell phenotype and exhaustion is as follows: First the 'Lymphocytes' were gated SSC-A:FSC-A to remove cellular debri; further the 'singlets' were gated FCSA: FSC_H and the live dead were dated in SSC-A: Near-infrared dye. The live cells are then gated for CD3+T cells (SSC-A:CD3) further these cells were gated for phenotypic distribution of T-cells based on CCR7 and CD45RA expression,(CCR7:CD45RA) showing Q2-TN ($CD45RA^{+}CCR7^{+}$), Q1-TCM ($CD45RA^{-}CCR7^{+}$), Q3-TEM (CD45RA-CCR7-), and Q4-TEMRA (CD45RA+CCR7-) subset as shown in supplementary figure 13d or CD27 and CD28 expression (CD27:CD28) where Q2-CD27+CD28+ were plotted as shown in figure 3d. The exhaustion profile of the T cells were also gated using PD1 and Lag-3(PD1:Lag-3) where Q2- PD1+Lag-3+ were plotted in Figure 3e.
Gating strategy for CAR T cell phenotype is as follows: First the 'Lymphocytes' were gated SSC-A:FSC-A to remove cellular debri; further the 'singlets' were gated FCS-A:FSC_H and the live dead were dated in SSC-A: Near-infrared dye. The live cells are then gated for CD3+T cells (SSC-A:CD3). The T cells were gated for CD19 CAR expression (SSC-A:GFP) or (SSCA: CD19-Tetramer) were plotted in Figure 3f, further these cells were gated for phenotypic distribution of T-cells based on CCR7 and CD45RA expression,(CCR7:CD45RA) showing Q2-TN ($CD45RA^{+}CCR7^{+}$), Q1-TCM ($CD45RA^{-}CCR7^{+}$), Q3-TEM (CD45RA-CCR7-), and Q4-TEMRA (CD45RA+CCR7-) subset as shown in Figure 3k or CD27 and CD28 expression (CD27:CD28) where Q2-CD27+CD28+ were plotted as shown in Figure 3j.
Gating strategy for CAR T cell phenotype and exhaustion post rechallenge cytotoxicity assay is as follows: First the 'Lymphocytes' were gated SSC-A:FSC-A to remove cellular debri; further the 'singlets' were gated FCS-A:FSC-H and the live dead were gated in SSC-A: Nearinfrared dye. The live cells are then gated for Jeko-mcherry tumor cells and CD3+T cells (Pecf-594-mcherry:CD3) and tumor cells were excluded by gating CD3-Pecf-594 mcherry+ and the T cells were gated using CD3+Pecf-594 mcherry- and were plotted as shown in Figure 4i. The T cells were gated for CD19 CAR expression (SSC-A:GFP) or (SSC-A:CD19-Tetramer), further these cells were gated for phenotypic distribution of T-cells based on CCR7 and CD45RA expression,(CCR7:CD45RA) showing Q2-TN ($CD45RA^{+}CCR7^{+}$), Q1-TCM ($CD45RA^{-}CCR7^{+}$), Q3-TEM (CD45RA-CCR7-), and Q4-TEMRA (CD45RA+CCR7-) subset as shown in Figure 4f or The exhaustion profile of the T cells were also gated using PD1 (SSC-A :PD1), Lag-3(SSC-A:Lag-3), Tim-3(SSC:A:Tim-3), TIGIT(SSC-A:TIGIT) as shown in supplementary figure 18(a,b,c) and plotted in Figure 4g.
Gating strategy for For analysis of the cells from spleen and bone marrow of the mice treated with T cells or CAR T cells expanded with T-Expand or Dynabeads is as follows; First the 'Lymphocytes' were gated SSC-A:FSC-A to remove cellular debri; further the 'singlets' were gated FCS-A:FSC-H and the live dead were gated in SSC-A: Near-infrared dye. The live cells are then gated for Jeko-mcherry tumor cells and CD3+T cells (Pecf-594-mcherry:CD3) and tumor cells were excluded by gating CD3-Pecf-594 mcherry+ and the T cells were gated using CD3+Pecf-594 mcherry- and were plotted as shown in supplementary figure 16. The T cells were gated for CD19 CAR expression (SSC-A:GFP) or (SSC-A:CD19-Tetramer) supplementary figure 19 and plotted in Figure 5d or CD4 and CD8 T cells using (CD4:CD8) and the CD8+ T cells were gated using CD4-CD8+ gate and CD4 T cells were gated using CD8-CD4+ gate as shown in supplementary figure 19 and plotted in Figure 5e. Further the CD137 expressing CAR T cells were gated using (SSC-A:CD137) as plotted in Figure 5f.

☒ Tick this box to confirm that a figure exemplifying the gating strategy is provided in the Supplementary Information.
